# The Duration of Antigen-Stimulation Significantly Alters the Diversity of Multifunctional CD4 T Cells Measured by Intracellular Cytokine Staining

**DOI:** 10.1371/journal.pone.0038926

**Published:** 2012-06-12

**Authors:** Daryan A. Kaveh, Adam O. Whelan, Philip J. Hogarth

**Affiliations:** TB Research Group, Animal Health and Veterinary Laboratories Agency (AHVLA), Surrey, United Kingdom; University of Cape Town, South Africa

## Abstract

The assessment of antigen-specific T cell responses by intracellular cytokine staining (ICS) has become a routine technique in studies of vaccination and immunity. Here, we highlight how the duration of *in vitro* antigen pre-stimulation, combined with the cytokine accumulation period, are critical parameters of these methods. The effect of varying these parameters upon the diversity and frequency of multifunctional CD4 T cell subsets has been investigated using a murine model of TB vaccination and in cattle naturally infected with *Mycobacterium bovis*. We demonstrate a substantial influence of the duration of the antigen pre-stimulation period on the repertoire of the antigen-specific CD4 T cell responses. Increasing pre-stimulation from 2 to 6 hours amplified the diversity of the seven potential multifunctional CD4 T cell subsets that secreted any combination of IFN-γ, IL-2 and TNF-α. However, increasing pre-stimulation from 6 to 16 hours markedly altered the multifunctional CD4 T cell repertoire to a dominant IFN-γ^+^ only response. This was observed in both murine and cattle models.

Whilst these data are of particular relevance to the measurement of vaccine and infection induced immunity in TB, more generally, they demonstrate the importance of the empirical determination of the optimum duration of the individual culture steps of ICS assays for any model. We highlight the potential significance of variations in these parameters, particularly when comparing data between studies and/or models including clinical trials.

## Introduction

The application of detecting antigen-specific cytokine production in individual T cells by intracellular cytokine staining (ICS) and flow cytometry has become a common and insightful technique in studies of immunity to infectious diseases. The simultaneous staining of three or more cytokines allows division of responder cells into subsets based upon their co-production profile and identifies cells of a ‘multifunctional/polyfunctional’ profile.

Such T cells have been strongly associated with immunity to HIV [1], [2]; influenza [3], [4]; leishmania [5] and tuberculosis (TB) [6], [7], [8], [9], [10], [11], [12]. Consequently, they are seen as a promising measure of vaccine efficacy [13] and are a commonly measured immunological parameter in clinical trials [14], [15], [16], [17], [18].

While ICS protocols have become relatively standardised [19], [20], especially in the context of minimising variability within clinical trials [21], [22], [23], one variable which is often not described is the duration of antigen pre-stimulation before a golgi transport inhibitor is added to the culture.

The identification of these functional responses relies upon an *in vitro* culture step to specifically re-stimulate the T cells with relevant antigen. During this step, a golgi transport inhibitor such as brefeldin A (BFA) or monensin is required to prevent secretion and allow accumulation of intracellular proteins to levels detectible by ICS. The choice of inhibitor is dependent on the proteins of interest; i.e. BFA for TNF-α detection and monensin for degranulation proteins [24].

When peptide antigens are used as the T cell stimulus the transport inhibitor of choice can be added at the start of the culture since they do not require endosomal processing [20]. However, when protein antigens are used, either as single recombinant proteins or complex mixtures, then a period of pre-stimulation is required before the addition of the transport inhibitor to allow sufficient antigen processing before disruption of intracellular transport mechanisms [25], [26], [27].

The optimal duration of this antigen pre-stimulation period most is likely to vary between different immunity models, antigens and culture conditions. It is therefore important that its duration be established empirically for each model. Inevitably, the duration of this pre-stimulation, will be a compromise between maximal antigen presentation, adequate analyte accumulation and the practicality of the culture schedule.

The actual effect that variations in the pre-stimulation duration have on the cytokine responses, and ultimately the definition of responder T cell subsets, will not be apparent until tested. Here, we sought to examine the effect of antigen pre-stimulation duration upon the antigen-specific CD4 T cell response in both a murine and bovine model of TB immunity. To this aim, we evaluated the cytokine responses elicited by *Mycobacterium bovis* bacille Calmette-Guèrin (BCG) vaccination in mice as well as those in cattle naturally infected with *M. bovis*. On making comparisons between using either short (2–6 hours) or overnight (16 hours) pre-stimulation periods, both models showed the same substantial differences between the responder cell cytokine repertoire detected after the different culture schedules. Our results demonstrate the potential significance of varying the culture duration parameters with regard to the results generated by the ICS analyses and the interpretation the data. Given the common use of ICS techniques, these data have important implications for the standardisation of such assays between laboratories.

## Results

### The effect of different culture schedules on antigen-specific murine CD4 T cell responses

**Figure 1:**
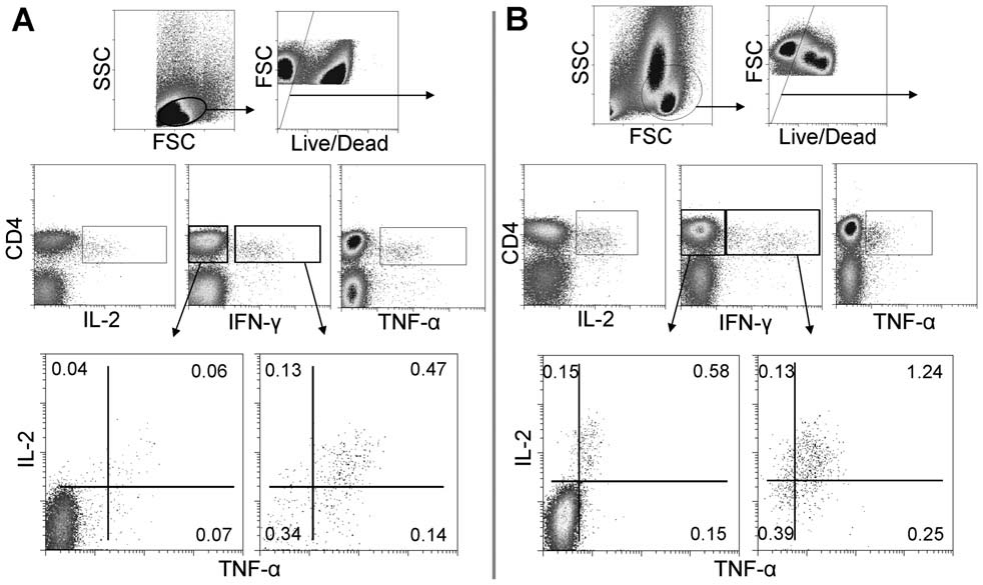
Identification of CD4^+^ T cells producing IFN-γ, IL-2 & TNF-α. Mouse splenocytes 6 weeks following a BCG immunization (A) and PBMC from cattle naturally infected *M. bovis* (B) were isolated, stimulated with antigen, stained by intracellular cytokine staining (ICS) and interrogated by flow cytometry. Plots were gated on live CD4^+^ lymphocytes and analyzed for all combinations of simultaneous IFN-γ, IL-2 and TNF-α productivity. Numbers indicate percentage of CD4^+^ cells in the seven individual cell subsets, data are representative of one of three independent experiments.

Splenocytes were isolated from mice immunised for six weeks with BCG and were cultured *in vitro* with a protein cocktail of 7 BCG derived antigens and subsequently interrogated by ICS and flow-cytometric analysis. Five independent culture schedules were compared in parallel. These differed by increasing lengths of antigen pre-stimulation period (from 0 to 16 hours) and/or the subsequent period of time after BFA addition (from 4 to 16 hours). Multicolour ICS analysis of the simultaneous production of IFN-γ, IL-2 and TNF-α by antigen-specific CD4 T cells, allowed for the subdivision of these cytokine producing cells into seven functional subsets, as demonstrated on a representative sample (Figure 1A). Samples from placebo control mice were analysed in parallel (data not shown) and any responses were subtracted from those of the BCG stimulations to provide vaccine specific responses.

**Figure 2:**
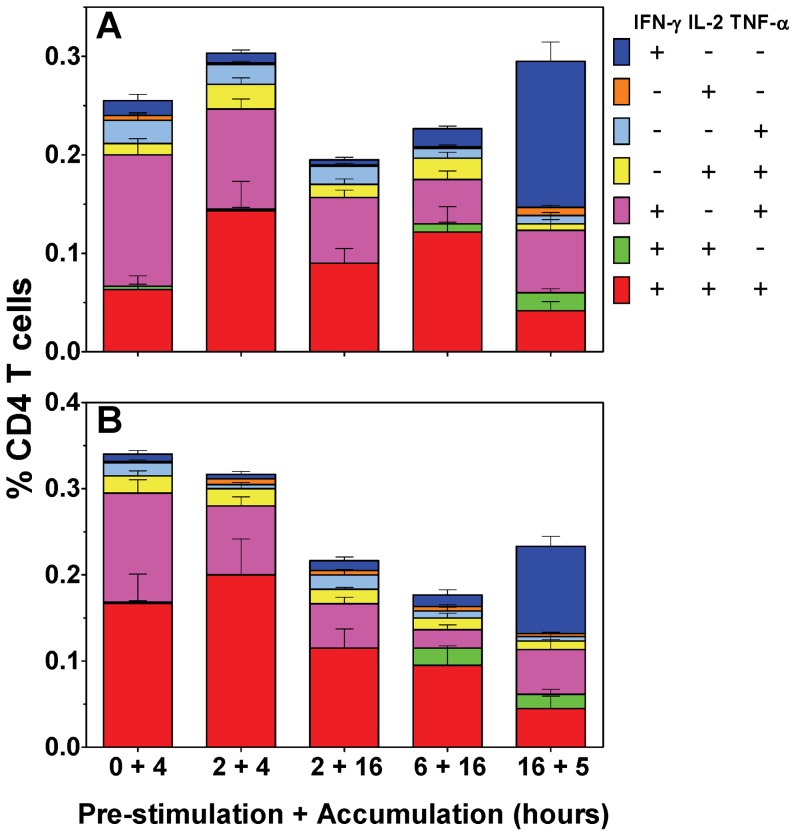
The effect of different culture schedules on antigen-specific murine CD4 T cell responses. Six weeks following immunization, spleen cells from BCG or placebo control vaccinates were isolated, stimulated with a BCG derived recombinant protein cocktail (A) or individual immunodominant peptide of Rv0288 (B), stained by intracellular cytokine staining (ICS) and interrogated by flow cytometry. Plots were gated on live CD4^+^ lymphocytes and analyzed for all combinations of simultaneous IFN-γ, IL-2 and TNF-α productivity. The stacked bar graphs represent the percentage of antigen-specific CD4^+^ cells in the seven individual cell subsets, data are representative of one of three independent experiments (n = 6). Bars represent the mean ± SEM.

Figure 2A shows the frequency of the CD4 T cell subsets induced by the different culture schedules. Adding the golgi inhibitor, BFA, at the start of antigen-stimulation induced two dominant populations of IFN-γ^+^TNF-α^+^ (0.13%) and triple positive multifunctional IFN-γ^+^IL-2^+^TNF-α^+^ (0.06%) cells and minor populations of all other subsets.

Antigen pre-stimulation for 2 hours prior to BFA addition, increased the frequency of multifunctional IFN-γ^+^IL-2^+^TNF-α^+^ cells to 0.14%, (ns.).

Further increasing the duration of BFA treatment from 4 to 16 hours, resulted in a statistically significant reduction in IFN-γ^+^TNF-α^+^ cells (0.07%, p<0.01 *vs.* no pre-stimulation). Increasing the duration of antigen pre-stimulation to 6 hours, revealed a small but distinct population (0.01%) of IFN-γ^+^IL-2^+^ cells.

Further increasing the antigen pre-stimulation period to 16 hours resulted in a significant drop from the maximal frequency of multifunctional IFN-γ^+^IL-2^+^TNF-α^+^ cells (0.04%, p<0.05 *vs.* 2+4 hours), but a concurrent significant gain in the frequency of single IFN-γ^+^ cells (0.12%, p<0.001 *vs.* all other conditions), IFN-γ^+^IL-2^+^ (0.02%, p<0.05, *vs.* all conditions) and IL-2^+^ (0.01%; p<0.05 *vs.* 2+4; 2+16 and 6+16 hours) cells.

The use of peptide rather than protein antigen may require different antigen processing mechanisms that could affect the results. This was evaluated by running parallel analysis using an immunodominant peptide of Rv0288, which is highly immunogenic in BCG immunised BALB/c mice [28], [29]

Figure 2B reveals that the distribution of the profiles of peptide induced CD4 T cell responses were less dependent on antigen pre-stimulation to those induced by the protein cocktail. The frequency of IFN-γ^+^IL-2^+^TNF-α^+^ cells was equivalent with no pre-stimulation to that following 2 hours of pre-stimulation (0.17% *vs.* 0.2%). Pre-stimulation with antigen did induce a reduction in frequency of IFN-γ^+^TNF-α^+^ cells (p<0.05 *vs.* 0+4 hours). Increasing pre-stimulation to 6 hours induced significantly greater frequencies of the rare IFN-γ^+^IL-2^+^ cells (0.02% *vs*. <0.002%, p<0.01 *vs.* 0+4, 2+4 or 2+16 hours). 16 hours pre-stimulation significantly decreased detection of multifunctional IFN-γ^+^IL-2^+^TNF-α^+^ cells to 0.05% from their maximal frequencies (p<0.05 *vs.* 0+4 hours; p<0.01 *vs.* 2+4 hours), but significantly increased the frequency of single IFNγ^+^ cells (0.1%; p<0.001 *vs.* all other conditions), while the population of IFN-γ^+^IL-2^+^ (0.02%; p<0.01 *vs.* 0+4, 2+4 or 2+16 hours) cells were still evident.

*The effect of different culture schedules on antigen-specific bovine CD4 T cell responses.*

In order to determine whether the change in multifunctional T-cell repertoire, as influenced by the duration of antigen stimulation, was specific to this murine model of BCG vaccination, PBMC isolated from cattle naturally infected with *M. bovis* were examined for PPD-B-specific CD4 T cell responses.

The application of ICS in the analysis of bovine cells has recently been enhanced by the ability to additionally detect IL-2 and TNF-α [30] together with the established detection of IFN-γ [31], [32]. Therefore, an equivalent parallel analysis was carried out comparing six independent culture schedules for their affect on the subsequent multicolour ICS analysis of the seven cytokine producing subsets of antigen-specific CD4 T cells. The responses from unstimulated parallel cultures were subtracted to generate these antigen-specific responses (Figure 1B).

**Figure 3:**
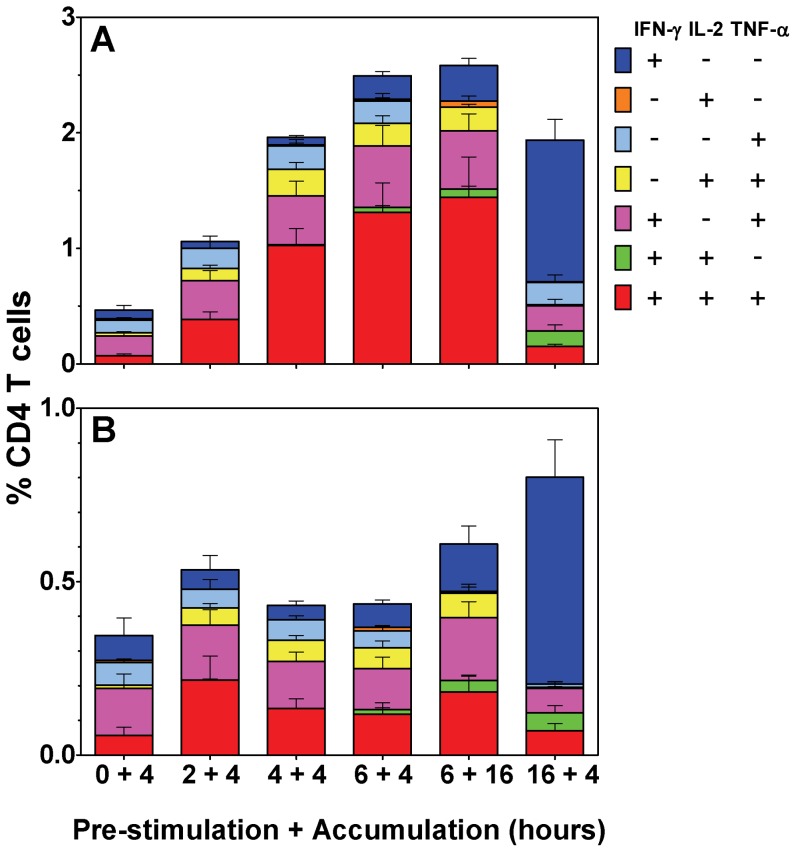
The effect of different culture schedules on antigen-specific bovine CD4 T cell responses. PBMC from cattle naturally infected with *M. bovis* were isolated, stimulated with PPD-B (A) or ESAT-6/CFP-10 peptide pools (B), stained by intracellular cytokine staining (ICS) and interrogated by flow cytometry. Plots were gated on live CD4^+^ lymphocytes and analyzed for all combinations of simultaneous IFN-γ, IL-2 and TNF-α productivity. The stacked bar graphs represent the percentage of antigen-specific CD4^+^ cells in the seven individual cell subsets, data is representative of one of three independent experiments (n = 6). Bars represent the mean ± SEM.

As shown in Figure 3A, the frequency of antigen-specific multifunctional IFN-γ^+^IL-2^+^TNF-α^+^ cells significantly increased with the duration of PPD-B pre-stimulation period, up to a maximum at 6 hours: rising from 0.07% to 0.38% at 2 hours (ns.); to 1.0% at 4 hours (p<0.05); and to 1.31% at 6 hours (p<0.001). Extending the BFA incubation by 12 hours only increased this response by 0.13%.

As observed in the murine system, extending the pre-stimulation period from 6 to 16 hours, resulted in a switch to a dominant IFN-γ^+^ cell response (1.23%; p<0.001 *vs.* all conditions), accompanied by a significant reduction in the frequency of IFN-γ^+^IL-2^+^TNF-α^+^ (0.15%, p<0.001) and IL-2^+^TNF-α^+^ cells (0.2% to 0.01%, p<0.05). This decrease in IFN-γ^+^IL-2^+^TNF-α^+^ cells also correlated with a significant increase in the detection of the rare IFN-γ^+^IL-2^+^ subset (0.13%; p<0.05 *vs.* pre-stimulations <6 hours).

As previously, parallel analyses were made using peptide antigens. Cultures were stimulated with a peptide pool mapping the *Mycobacterium bovis* immunodominant proteins ESAT-6 and CFP-10 [33]. As seen in Figure 3B, and in contrast to protein antigen stimulation; peptide pre-stimulation of less than 16 hours duration failed to induce a significant increase in the frequency of multifunctional IFN-γ^+^IL-2^+^TNF-α^+^ cells. Interestingly, the pre-stimulation of cells with peptide for 16 hours induced equivalent responses to those observed with protein antigen.

### The effect of different culture schedules on the viability of CD4 T cells

Another important outcome that may be influenced by the culture schedule used is the viability of the cells post-culture. Cell culture for ICS inherently induces a population of dead cells, which is likely exacerbated by the toxic effect of BFA. The use of fixable viability stains, reviewed in [34], [35], allow for the exclusion of dead cells from the analysis (seen in figure 1) to reduce non-specific staining artefacts. Here, the affect on the viability of the CD4 T cell population was analysed in the various parallel culture schedules.

**Figure 4:**
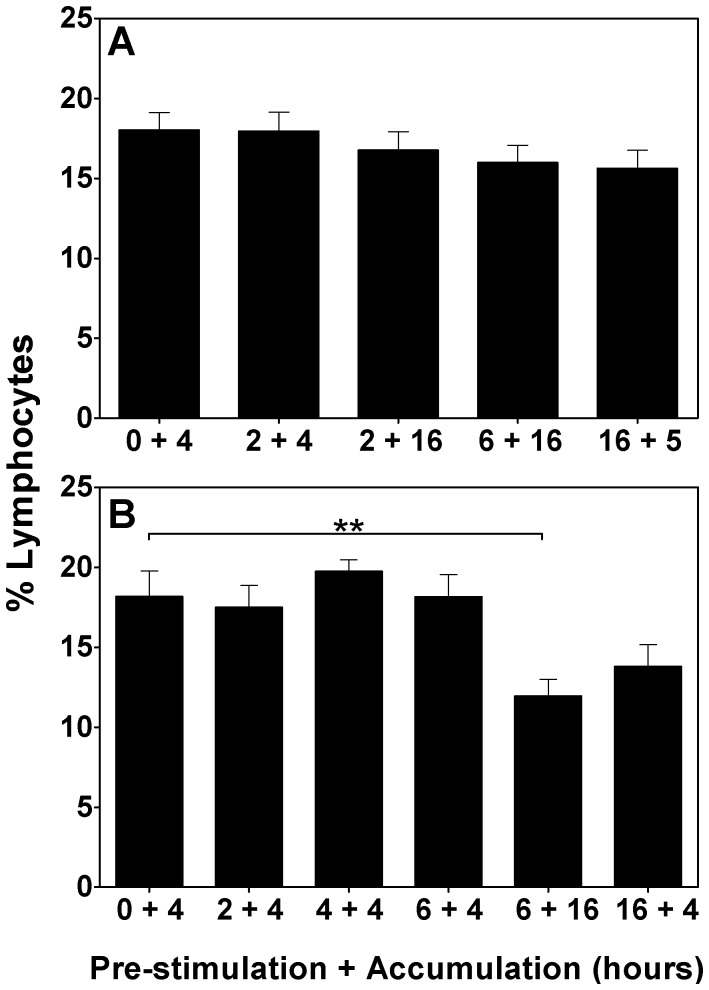
The effect of different culture schedules on the viability of CD4 T cells. Mouse splenocytes 6 weeks following a BCG immunization (A) and PBMC from cattle naturally infected with *M. bovis* (B) were isolated, stimulated with protein cocktail or PPD-B, respectively, stained by intracellular cytokine staining (ICS) and interrogated by flow cytometry. Graphs represent the percentage of live CD4^+^ lymphocytes detected in the analysis, as determined by the inclusion of a LIVE/DEAD fixable Violet dead cell stain. Data are representative of one of three independent experiments (n = 6). Bars represent the mean ± SEM; **p<0.01; ANOVA.

The frequencies of viable CD4 T cells in the murine lymphocyte cultures did not significantly differ between stimulation schedules (figure 4A).

In the bovine lymphocyte culture, however, stimulation schedules involving culture for over 20 hours in total resulted in a decline of viable CD4^+^ T cells (figure 4B), irrespective of the duration of BFA incubation. 6 hours pre-stimulation followed by 16 hours of BFA resulted in a 33% decline in viability (p<0.01 *vs.* no pre-stimulation) whilst 16 hours pre-stimulation followed by 4 hours of BFA resulted in a 23% drop (ns.).

## Discussion

Here, we have investigated the effect of altering the duration of the *in vitro* culture parameters on the ICS analysis of the co-production of IFN-γ, IL-2 and TNF-α by antigen-specific multifunctional CD4 T cells. Our rationale for undertaking this investigation was to ensure the development of optimal conditions for inducing and measuring multifunctional responses. When developing such methodologies, the practicality of the protocol is an important consideration especially if it is to be applied on a routine basis. We therefore examined the length of time used for the antigen pre-stimulation period, and that of the cytokine accumulation period after BFA addition, in murine and bovine CD4 T cells. Schedules that could be routinely used in the laboratory were investigated and the biological consequences of these schedules were assessed.

Evaluation of the cytokine response of murine and bovine antigen-specific CD4 T cells to protein antigens demonstrated that extending antigen pre-stimulation to 16 hours (i.e. an overnight stimulation), followed by 4/5 hours of BFA incubation, resulted in a response dominated by IFN-γ^+^ CD4 T cells. This was accompanied by an increase in the total diversity of the response and a reduction in the multifunctional IFN-γ^+^IL-2^+^TNF-α^+^ population, compared to the use of shorter pre-stimulation periods.

BFA is toxic during long culture periods, presumably due to the disruption of intracellular transport for more than a few hours. Here, the frequency of bovine, but not murine, viable CD4 T cells did decrease with the two longest culture periods, but this was independent of the length of the BFA incubation. Importantly, this decrease was not accompanied by a decline in the total antigen-specific population.

Although the data from the murine and bovine models are largely complimentary, one major difference in response kinetics could be seen. The detection of bovine responder cells was clearly augmented by the use of longer pre-stimulation periods with protein antigen, as the frequency of IFN-γ^+^IL-2^+^TNF-α^+^ cells increased with duration of pre-stimulation, to a maximum at 6 hours. However, in both models, this dependence on antigen pre-stimulation for optimal multifunctional cell frequency was abrogated when peptide antigen was used. This suggests that our murine response may be better measured using the peptide antigen, whilst in this bovine system there is a clear advantage in using the protein antigen combined with pre-stimulation. This may be due to the murine analysis being dominated by a single clonal response to TB10.4, whilst the naturally infected cattle may have responded to more antigens found in PPD than in the ESAT-6/CFP-10 peptide pool.

This difference in response kinetics could be a consequence of several factors. For example: it could be due to host differences between mice and cattle; be due to the disparity of splenocytes with PBMCs; or be due to differences in antigen-specific cell populations in the two different models of immune response. One significant factor, is that the bovine model did not use antibodies to augment co-stimulatory activation of the T cells, such as the anti-CD28 used in the murine analysis or anti-CD49d used additionally by others. These increase the frequency of detectible antigen-specific responding cells and depending on their degree of utilisation, can have a substantial influence [36].

The length of pre-stimulation will affect the potential for antigen processing/presentation, whilst the overall culture period will increase the probability for antigen-specific antigen presenting cell induced stimulation and activation of T cells, but explanations for the significant effects observed here are not clear.

Effector cytokines released by antigen experienced CD4 T cells are produced *de novo* [37], rather than being stored as preformed proteins ready for immediate release. The preformed mRNA transcripts found within these cells [38], [39], [40] would permit for relatively rapid cytokine production, as illustrated by their detection in the shorter culture periods. The increased detection of additional functional subsets on lengthening the pre-stimulation period, may have been due to differences in the de novo production of these cytokines by different populations of CD4 T cells. These could be caused by several mechanisms. The dynamics of production of the individual cytokines could differ, for instance, the secretory pathway of TNF-α has been shown to be distinct from that of IL-2 and IFN-γ [41]. Alternatively, the length of time over which these individual cytokines are produced could differ; more sustained IFN-γ than IL-2 and TNF-α production, could explain the observed shift in the balance from an increase in functional diversity to a predominantly IFN-γ^+^ response, when the pre-stimulation period was increased from 6 to 16 hours.

Nomura and colleagues compared the effect of lengthening ICS culture pre-stimulation periods up to 20 hrs in the optimization of detecting whole blood responses to CMV [42]. Although multifunctionality was not assessed, the study concurred with a substantial decrease in the detection of IL-2^+^ and TNF-α^+^ CD4 T cells in stimulations of 16 hours.

Previous work analysing CD40 ligand (CD154), a marker of the antigen-specific activation of CD4 T cells, revealed that the frequency of detectible activated cells increased until at least 6 hours of stimulation [43]. With evidence that CD154 is stored preformed in secretory lysosomes [44], this delay in its expression by some CD4 T cells is likely to be limited by the time needed for an activating encounter with presented antigen. Additionally, detection of these cells has also been shown to be influenced by the cell density used in the culture [45].

It must be considered that such ICS analyses are a snapshot of an *in vitro* initiated response and it is difficult to determine which functional representation most accurately reflects the *in vivo* situation. In the absence of such knowledge, we considered that the detection of a combination of the greatest diversity and frequency of functional subsets would be most prudent.

The identification of multifunctional T cells by ICS and their associations with protective immunity [1], [5] has resulted in a multitude of studies investigating these cells. Their measurement has become an established and central technique in many laboratories studying immunity to infection. Therefore, our demonstration of how sensitive this technique is to experimental conditions is a crucial finding, with wide ranging implications.

Evaluating the quality of an immune response can be challenging if it is unclear what constitutes a superior response, especially when ICS analyses are not consistent and representative between comparisons. ICS culture durations do vary between studies, but investigators should establish exact optimal durations for their studies as these could be dependant on specifics of the culture, such as the T cells themselves, cell milieu composition and the antigens used for stimulation. Where cross lab comparisons of results are made, methodological parity is necessary, especially in clinical trials, as discussed by Hannekom and colleagues [46].

In summary, our data have shown the substantial influence that alterations in the duration of the ICS culture pre-stimulation period and cytokine accumulation period can have on the cytokine repertoire of antigen-specific CD4 T cells. Therefore, this should be taken into account when deciding upon what protocol to use and when interpreting and comparing ICS data between studies.

## Materials and Methods

### Animals

All animal procedures were approved by the Animal Health and Veterinary Laboratories Agency (AHVLA) Animal Use Ethics Committee (UK Home Office PCD 70/6905), and performed under appropriate personal and project licenses within the conditions of the Animals (Scientific Procedures) Act 1986. All animals were housed in appropriate biological containment facilities at the AHVLA.

Female BALB/c mice were obtained from SPF facilities at Charles River UK Ltd. and used at 8 weeks of age. To investigate immune responses in cattle naturally exposed to *M. bovis*, tuberculin skin-test reactor cattle were recruited from UK farms with a confirmed history of bovine TB and infection status of these cattle was confirmed by the presence of TB lesions at post mortem examination, as previously described [30]. Non-infected control cattle were obtained from UK TB-free herds located in non-endemic areas.

### Mycobacteria

The vaccination strain used was the human vaccine *M. bovis* BCG Danish 1331, prepared as per manufacturer's instructions (SSI, Denmark).

### Immunization

Mice were immunized with a single intradermal (i.d.) injection in the tail-base of 2×10^5^ CFU of BCG. Placebo control mice were immunized with phosphate buffered saline (PBS). Six weeks later mice were euthanized and spleens harvested.

### Cell isolations and stimulations

Mouse spleen cells were prepared as described previously [9]. Cells were cultured at 5×10^6^/ml with 1 µg/ml anti-CD28 (BD biosciences, UK) plus a pool of seven recombinant protein antigens (Rv1886c, Rv0251, Rv0287, Rv0288, Rv3019c, Rv3763, Rv3804c) as described previously [9], each protein at a final concentration of 2 µg/ml. Alternatively, cells were stimulated with the immunodominant peptide antigen (16mer: a.a.73–88) of Rv0288 (Pepscan, the Netherlands) at a final concentration of 2 µg/ml. Samples from placebo control mice were analysed in parallel and any responses were subtracted from those of the BCG stimulations to provide vaccine specific responses.

Bovine peripheral blood mononuclear cells (PBMC) were prepared as described previously [47]. Cells were cultured at 2×10^6^/ml in the presence of 1∶100 dilution of Lelystad bovine tuberculin purified protein derivative (PPD-B, Prionics, Switzerland). Alternatively, cells were stimulated with a cocktail of peptides derived from the mycobacterial antigens ESAT-6 and CFP-10. This peptide cocktail was prepared using 21 peptides (16mers with 8-a.a. overlap) to provide complete mapping of the full protein sequences of ESAT-6 and CFP-10 (Pepceuticals Ltd., United Kingdom). The peptide cocktail was used at a final concentration of 5 µg/ml per peptide component. The responses from unstimulated parallel cultures were subtracted to generate antigen-specific responses.

### Antibody staining and Flow cytometry

Murine spleen cells were pre-stimulated for 0 to 16 hours at 37°C/5% CO_2_ before addition of BFA (Sigma) (10 µg/ml) and further culturing for 4 to 16 hours. Cells were washed and surface stained with CD4–APC-H7 (BD bioscience) and LIVE/DEAD fixable Violet dead cell stain (Invitrogen, UK). Subsequently, the cells were washed, treated with Cytofix and Perm Wash (BD biosciences), according to manufacturer's instructions, to intracellularly stain with IFN-γ-APC (BD biosciences), IL-2–PE-Cy7 and TNF-α-FITC (both eBioscience, USA).

Cattle PBMC were stimulated as above, washed and surface stained with CD4-APC-AlexaFluor 750 (AbDSerotec; custom conjugated with APC-AlexaFluor 750 by Beckman Coulter Inc. (Marseille, France)) and LIVE/DEAD fixable Violet dead cell stain. Subsequently, the cells were washed, treated with Cytofix and Perm Wash and intracellularly stained with IFN-γ-PE, TNF-α-AlexaFluor 488 and IL-2-DyLight649 (all AbD Serotec) [30].

Cells were analysed using a CyAn ADP analyser and Summit software (Beckman Coulter, USA), on acquisitions of a minimum of 100,000 live lymphocytes.

### Statistical analyses

All data were analyzed using the Graphpad Instat 3 statistical package (Graphpad, USA). Data were analyzed by ANOVA with Tukey-Kramer (ICS) or Dunnett post test (viability).
